# Coupled Optical and Electrochemical Probing of Silver Nanoparticle Destruction in a Reaction Layer

**DOI:** 10.1002/open.201800048

**Published:** 2018-05-23

**Authors:** Christopher A. Little, Christopher Batchelor‐McAuley, Kamonwad Ngamchuea, Chuhong Lin, Neil P. Young, Richard G. Compton

**Affiliations:** ^1^ Physical and Theoretical Chemistry Laboratory Oxford University South Parks Road Oxford OX1 3QZ United Kingdom

**Keywords:** dissolution, nanoimpacts, nanoparticles, optical, silver

## Abstract

The oxidation of silver nanoparticles is induced to occur near to, but not at, an electrode surface. This reaction at a distance from the electrode is studied through the use of dark‐field microscopy, allowing individual nanoparticles and their reaction with the electrode product to be visualized. The oxidation product diffuses away from the electrode and oxidizes the nanoparticles in a reaction layer, resulting in their destruction. The kinetics of the silver nanoparticle solution‐phase reaction is shown to control the length scale over which the nanoparticles react. In general, the new methodology offers a route by which nanoparticle reactivity can be studied close to an electrode surface.

## 1 Introduction

The reactivity and toxicity of silver nanoparticles (AgNPs) are intimately associated with their redox behavior. The arguably increased toxicity of nano‐Ag compared with the bulk metal has been linked to the oxidative dissolution^1^ of the AgNPs, with their greater potency attributed to the coupled formation of reactive oxygen species such as hydrogen peroxide.^2^ Consequently, in order to probe this redox chemistry, the behavior of NPs has been well studied at interfaces by using conventional electrochemical techniques. Commonly, research has employed stripping voltammetry to investigate varying the influence and apparent size effects on NP oxidation,^3^, ^4^ how the voltammetric response varies in relation to the NP surface coverage,^5^ the influence of NP surface charge,^6^ and the interaction with the underlying substrate.^7^ Even for sizes above the weak quantum confinement limit, alterations of the redox properties of the AgNPs are anticipated to occur as a result of the increased surface tension of the metal with decreasing particle size, as first explored by Plieth.^8^

AgNPs exhibit a strong plasmon peak at approximately 400 nm and, hence, both strongly absorb and scatter light near this wavelength. The position of this plasmon peak is sensitive to the NP size, shape, local dielectric environment and has also been shown to be sensitive to the NP potential. The latter was studied by using large ensembles of small Ag particles impacting at a transparent rotating disc electrode.^9^ Considering that in the Rayleigh scattering limit the intensity of the light scattered varies with the sixth power of the NP radius,^10^, ^11^ under dark‐field illumination, individual particles with diameters greater than approximately 20 nm can be readily individually visualized by using a conventional optical microscope. Hence, this technique allows the study of both the formation^12^ and redox chemistry^13^ of individual NPs. The development of combined optoelectrochemical setups has enabled the study of individual particles at electrochemical interfaces. These studies focused on nanomaterial affixed to or impacting an electrochemical interface.^14^ Although spectroscopic information is attainable, much of the work in the literature has focused on the intensity of the scattered light from individual particles and its alteration during the course of their oxidation. This allows the processes to be dynamically monitored and yields potential insight into the reaction kinetics at the nanoscale.^15^, ^16^, ^17^ Further development of holographic techniques to enable NP tracking in three dimensions has enabled the simultaneous in situ tracking of particles adjacent to and at an electrochemical interface.^18^, ^19^, ^20^ Elsewhere, the tracking of NP trajectories is a well‐established technique for studies into areas including single NP growth or single NP reactions using a single NP spectrometer.^21^, ^22^, ^23^

In biological and environmental contexts, NPs are isolated from any metallic conductive surface when undergoing redox reactions. Owing to their ability to accept and donate electrons in the solution phase, metallic NPs can be viewed as small isolated electrochemical systems, where their potential is defined by the local electrochemical environment. This view was expressed most clearly by Henglein who referred to such materials as ‘colloidal microelectrodes’.^24^, ^25^ Consequently, there is a desire for methods that enable the investigation of NP redox behavior in the solution phase further away from an electrode. Historically, such work has focused on the study of large ensembles of NPs in the solution phase by employing techniques such as UV/Vis spectroscopy. Nanoparticle UV/Vis spectroscopy is sensitive to a wide range of factors; however, the interpretation, quantification and delineation of such altered spectra can be challenging for such an ensemble measurement, owing to variations arising as a result of dissolution, agglomeration or a change in the capping agent.

An electrochemical reaction at an interface leads to an alteration in the concentration of redox species in the diffusional vicinity of the electrode. The product of the electrode reaction may diffuse away from the electrode surface, altering the local electrochemical environment and creating a concentration profile. In the presence of a suitable reagent, the electrode product may undergo further reaction in the solution phase. The region over which such a solution phase reaction occurs as driven by the interface, is termed the reaction layer. Recent work has also sought to exploit this reaction layer to enable heterogeneity in electrode activity to be visualized.^26^, ^27^ Recognizing that solution‐phase‐based NPs may intrinsically act as small isolated redox systems, this work seeks to explore the ability to controllably drive a reaction at a NP situated within the diffusion layer of an electrode.

This work opens a new route by which the solution‐phase dynamics of individual NPs may be studied in a stochastic manner, yielding information regarding their activity. The visualization of the individual NP reactivity is enabled through the coupling of dark‐field microscopy and electrochemistry. First, the electrochemical response of the AgNP system is investigated to identify and understand conditions under which the electrochemical reaction is seemingly halted. Second, the use of a combined optoelectrochemical cell reveals the origin of the cessation to be the oxidation of the NPs as a result of their reaction with a molecular product (bromine) of oxidation at the electrode. Third, theoretical models to explain the observed solution‐phase reaction and the length scales involved are developed. Finally, experiment and theory are compared, yielding insight into the complexities of the reaction.

## 2 Results and Discussion

This section commences by studying the oxidation of AgNPs in various electrolytes using the nanoimpact method. This is achieved through cyclic voltammetry and double potential step chronoamperometry at a microdisc electrode, demonstrating how applying high potentials to a suspension of NPs can temporarily cease the transient oxidative response. Next, we consider and quantify the optoelectrochemical response of the AgNPs in a thin‐layer optical cell with consideration of why the concentration of the AgNPs is depleted in aqueous solution at distances of up to around 100 μm from the electrode in the optical field. Last, we discuss and model the possible physical processes occurring in the oxidation reaction, and their interpretation.

### 2.1 Nanoimpacts of AgNPs

**Figure 1:**
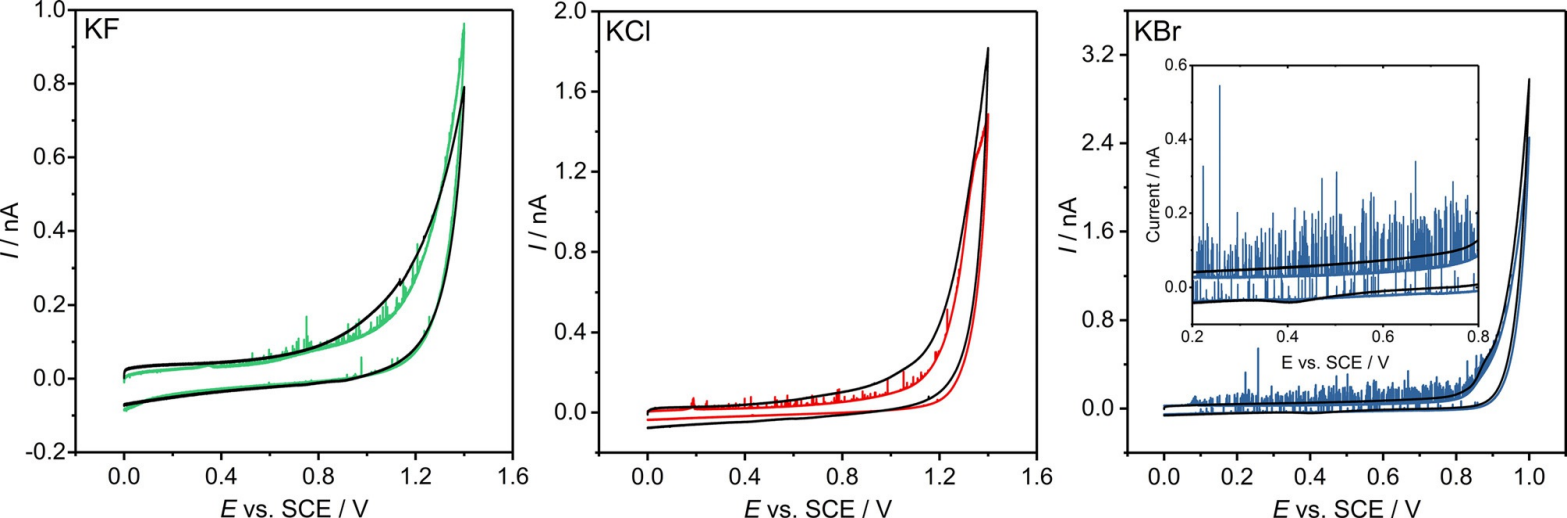
Representative cyclic voltammograms in the presence (colored lines) and absence (black lines) of 12 pm AgNPs in 20 mm KF, KCl, and KBr (left to right) supporting electrolytes. All measurements made at a carbon microdisc working electrode (16.5 μm radius) and a scan rate of 10 mV s^−1^. Inlay depicts the potential range 0.2 V to 0.8 V in more detail for KBr supporting electrolyte, displaying the reduction in frequency of oxidative spikes on the backward scan.

Figure 1 presents representative cyclic voltammograms in the presence (12 pm) and absence of AgNPs in different halide‐based electrolytes (KF, KCl or KBr; 20 mm). Without AgNPs present, no oxidative spikes are observed. In the presence of solution‐phase NPs and at potentials above a threshold value, small but distinct transient oxidative features are observed in the voltammetric response. These spikes in current relate to the arrival and oxidation of individual AgNPs at a suitably potentiostated electrochemical interface. Transport of the NPs to the interface occurs by virtue of their Brownian motion, albeit subjected to local surface‐hindered diffusion^28^, ^29^ and, hence, the oxidative events are observed to be a random and stochastic process. The potential at which these spike‐like features onset is sensitive to the identity of the halide; the potential increases from bromide to chloride to fluoride. This halide sensitivity has been discussed in previous work and, in part, reflects the alteration in the oxidation potential driven by complexation of the formed Ag^I^ ions.^30^

Also observable in the voltammograms are small oxidative waves that onset at 0.32, 0.18 and 0.09 V (vs. SCE) in fluoride, chloride and bromide, respectively. The magnitude of these waves is sensitive to the duration that the electrode has been immersed in the NP suspension. The Supporting Information (Section S4) presents representative voltammograms for immersion times of 10, 30 and 60 s prior to running the scans, as well as the integrated charges for the corresponding “stripping” wave. The magnitude of the wave is clearly shown to increase with the immersion time, and the total charge passed under this wave is comparable to the expected charge passed assuming irreversible NP adsorption limited by a steady‐state diffusional flux to the electrode. Consequently, it is concluded that these small “stripping” waves are associated with the oxidation of NPs pre‐adsorbed onto the electrochemical interface, indicating that the adsorption of the AgNPs occurs in the absence of the electrode being under potentiostatic control.

At potentials higher than approximately 0.8 V, in both the presence and absence of NPs, the oxidative breakdown of the solvent/electrolyte occurs. The onset of this process is also sensitive to the identity of the halide electrolyte; a current density of 50 μA cm^−2^ occurs at 1.27, 1.24 and 0.89 V (vs. SCE) in the presence of 20 mm fluoride, chloride and bromide, respectively. In the presence of fluoride and noting the standard electrode potential for the F^−^/F_2_ redox couple to be +2.87 V vs. SHE,^31^ it is reasonable that the increase in the current is solely the result of the oxidation of the solvent at the carbon interface. However, the notable shift in the onset of this oxidative feature in the presence of bromide strongly indicates that the process relates to bromide oxidation, where the standard potential for the Br^−^/Br_2_ redox couple is +1.07 vs. SHE.^31^ Questions regarding the exact chemical product of this oxidative process will be dealt with later in the text. Finally, in the case of chloride (for Cl^−^/Cl_2_, *E* ^o^=+1.36 vs. SHE) it is probable that both the oxidation of the solvent and the chloride could contribute at these high electrode potentials. At carbon electrodes, the balance between the rates of these two processes is known to be sensitive to a number of experimental factors.^32^

**Figure 2:**
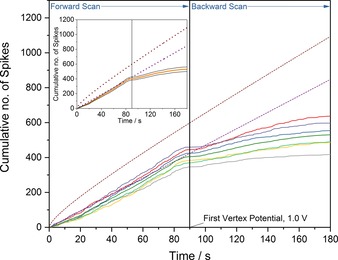
Cumulative number of spikes recorded as a function of time with 12 pm AgNPs in 20 mm KBr (solid lines). Also depicted are the theoretically predicted cumulative number of spikes derived from the integrated Shoup–Szabo Equation (brown dashed line) and the predicted number derived assuming a steady‐state flux (purple dashed line). Inlay displays the mean number of cumulative spikes from the seven voltammograms (orange line) with the associated standard error (grey lines).

In all three voltammograms (Figure 1 a–c), the most strikingly significant feature is the observed decrease in the frequency of the oxidative collisions on the reverse scan of the voltammogram. Taking bromide as an example, this reduction in frequency can be readily visualized by considering the cumulative number of spikes as a function of time (as opposed to the applied potential). Figure 2 presents the cumulative number of impacts for six cyclic voltammograms of 12 pM AgNPs in 20 mm KBr with the inlay depicting the average and subsequent standard error of these six experiments. The used experimental scan rate is 10 mV s^−1^ and the time origin (*t=*0) is taken as the potential of the stripping wave where the spike‐like oxidative features are observed to onset. The time at which the voltammogram reaches the vertex potential of 1.0 V vs. SCE has been marked on the plot. A notable feature of Figure 2 is the marked decrease in the rate of NP arrival at the electrochemical interface once the electrode has been swept to an oxidizing potential. Figure 2 also portrays the theoretically predicted number of cumulative spikes, as given by the integrated form of the Shoup‐Szabo equation^33^, ^34^ (neglecting near‐wall hindered diffusion)^28^, ^29^ and from the assumption of a steady‐state flux to the electrode. The method used in calculating these values has been described elsewhere,^30^ and leads to an AgNP diffusion coefficient of 9.8×10^−12^ m^2^ s^−1^ and a steady‐state flux of 4.7 collisions s^−1^.

From the inlay of Figure 2, the mean cumulative number of spikes observed experimentally closely follows the predicted steady‐state value until just before the first vertex potential. The observation that the values are, at times less than 80 s, lower than predicted by the integrated Shoup–Szabo equation (brown dashed line) can be rationalized by recognizing that the NPs accumulate irreversibly on the electrode before the experiment starts, leading to their depletion in the diffusion layer. This is directly evidenced by the presence of the anodic stripping peaks in the voltammetry (see Section S4 of the Supporting Information for further details). Furthermore, when individual NPs are located near to the electrode surface, their diffusion coefficients become anisotropic and reduced, significantly slowing their motion. This near‐wall hindered diffusion will further reduce the observed number of cumulative spikes in comparison to the predicted values from the integrated Shoup–Szabo equation.^28^, ^29^ Consequently, the initial higher NP flux predicted by the Shoup–Szabo equation is not reached, owing to prior depletion of the NP concentration adjacent to the electrode surface.

At times greater than 80 s in Figure 2, the flux of NPs to the electrode surface is significantly below that predicted by the steady‐state flux (purple dashed line). Moreover, as shown in Section S5 of the Supporting Information, this decrease in the NP flux is not simply related to the consumption of the NPs *at* the electrode surface or caused by the decreasing electrode potential on the reverse scan. Briefly, in the case of cyclic voltammograms performed in the same NP suspension with multiple scans but with a lower first vertex potential (0.3 V vs. SCE), there is no drop off in the frequency of oxidative collisions on the reverse scans, with the cumulative number of spikes observed closely following the steady‐state flux predicted value, as shown in Figure S5.

For all seven voltammograms shown in Figure 2, just before the first vertex potential, no spikes are observed for a short period of time. The mean “pause” in the observation of spikes is found to be 3.3±0.6 s and occurs at potentials greater than 0.983±0.003 V. As shown in Figure 2, at longer times on the back scan of the voltammogram (following the first vertex potential), the impacts do return; however, their frequency falls to significantly lower rates than the expected steady‐state value. Moreover, the area under each spike relates to the charge transferred in a single oxidative collision. The mean charges for spikes on the forward and back scans are 0.402±0.006 pC and 0.274±0.007 pC respectively, corresponding to an average reduction of around 32 % in the size of the individual nano‐event. This suggests that the NPs observed on the back‐scan tend to be smaller in size. The size distributions are presented in the Supporting Information (Section S6). Several possible physical interpretations of this decreased frequency may be described. First, the product of bromide oxidation at the higher potentials may react with or destroy the AgNPs within the diffusion layer around the electrode, leading to a reduced frequency of impacts. Second, the product of the solvent breakdown may induce forced mass transport of the AgNPs away from the electrode surface as resulting from possible diffusio‐electrophoretic processes.^19^ Finally, the oxidation of bromide at higher potentials may lead to fouling or temporary passivation of the electrode surface, hence blocking the electrode to silver oxidation.

### 2.2 Nanoparticle Dynamics in an Optoelectrochemical Cell

**Figure 3:**
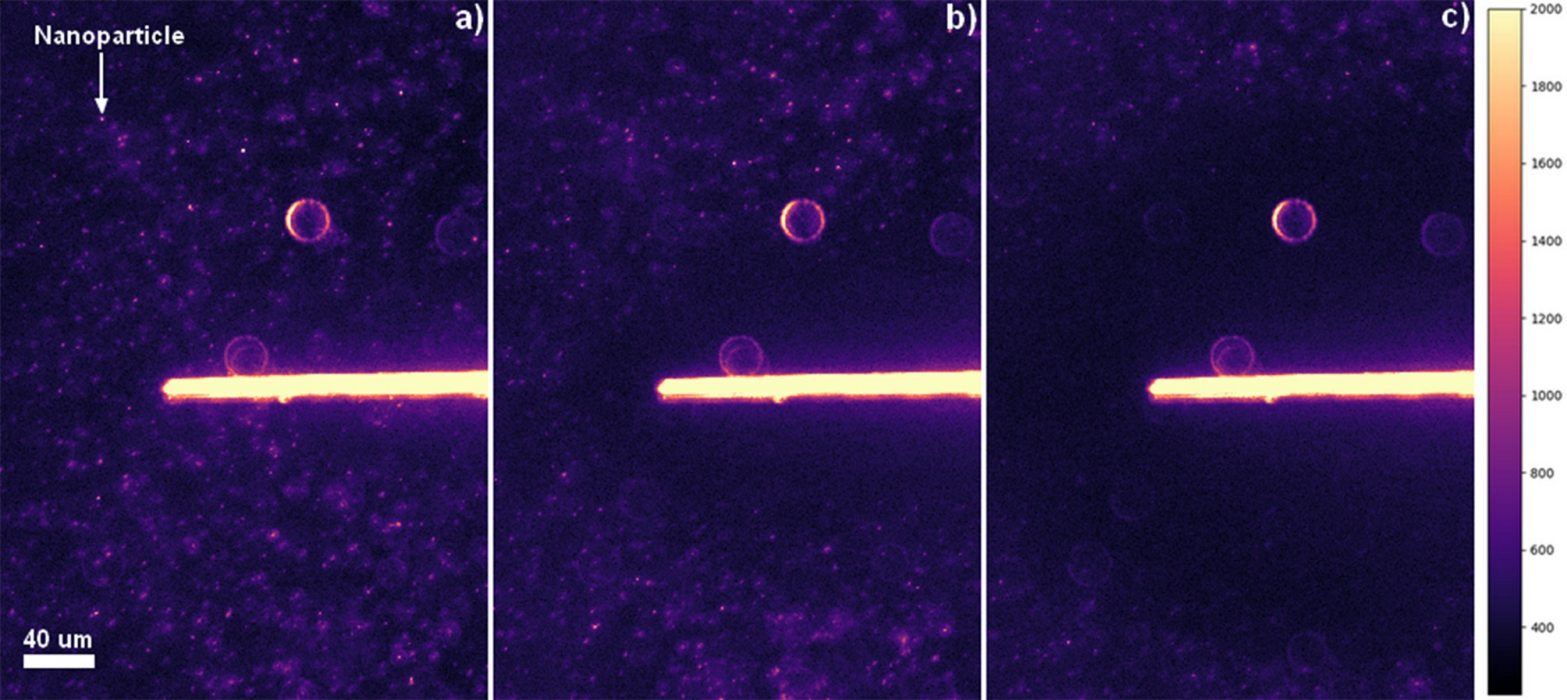
Representative dark‐field microscopy images of a carbon fiber wire immersed in a suspension of 1.2 pm AgNPs in 20 mm KBr under 20 × magnification. Images at a) 0 s, b) 3.1 s, and c) 14.1 s during the double step chronoamperometry. The images are falsely colored using a magma color map, with the intensity characterized by the scale shown on the right.

To clarify the physical origin of the observed decrease in the NP oxidative flux at the electrode surface, an optoelectrochemical cell was developed to allow visualization of the NPs in the vicinity of a carbon fiber electrode. Section S3 of the Supporting Information depicts a schematic of the cell design used, which employs a carbon fiber cylinder microelectrode. Plasmonic materials such as AgNPs are highly scattering at optical wavelengths and, hence, dark‐field illumination enables the NPs present in solution to be visualized as diffraction‐limited features in the optical field. Representative microscopy images of the optoelectrochemical cell are presented in Figure 3, whereby the carbon fiber electrode interface is clearly observed as the large scattering feature in the center of the optical field. In the solution phase, the NPs can be observed as small scattering features and, during the optical experiments, these can be seen to move. An example of these scattering features is labelled in Figure 3 a. Such NPs have previously been characterized by NP tracking analysis, using a Nanosight microscope, and were observed to have mobilities consistent with their hydrodynamic radii. The average distance a NP moves in 0.1 s is equal to (2*Dt*)^0^⋅^5^=1.4 μm, comparable with the distances they are observed to move between frames when imaged optically at 10 fps. Also visible in Figure 3, are several larger individual higher intensity features that remain constant throughout the experiment, resulting from the scattering of light from out‐of‐focus defects on the surface of the glass. Owing to the scattering of light from the carbon fiber itself, NPs can be visualized *near to*, but—for the present experimental setup—not *at*, the carbon fiber surface. Consequently, no NPs may be visualized within approximately 13.5 μm of either side of the center of the wire, compared to the true radius of the wire of 3.5 μm.

**Figure 4:**
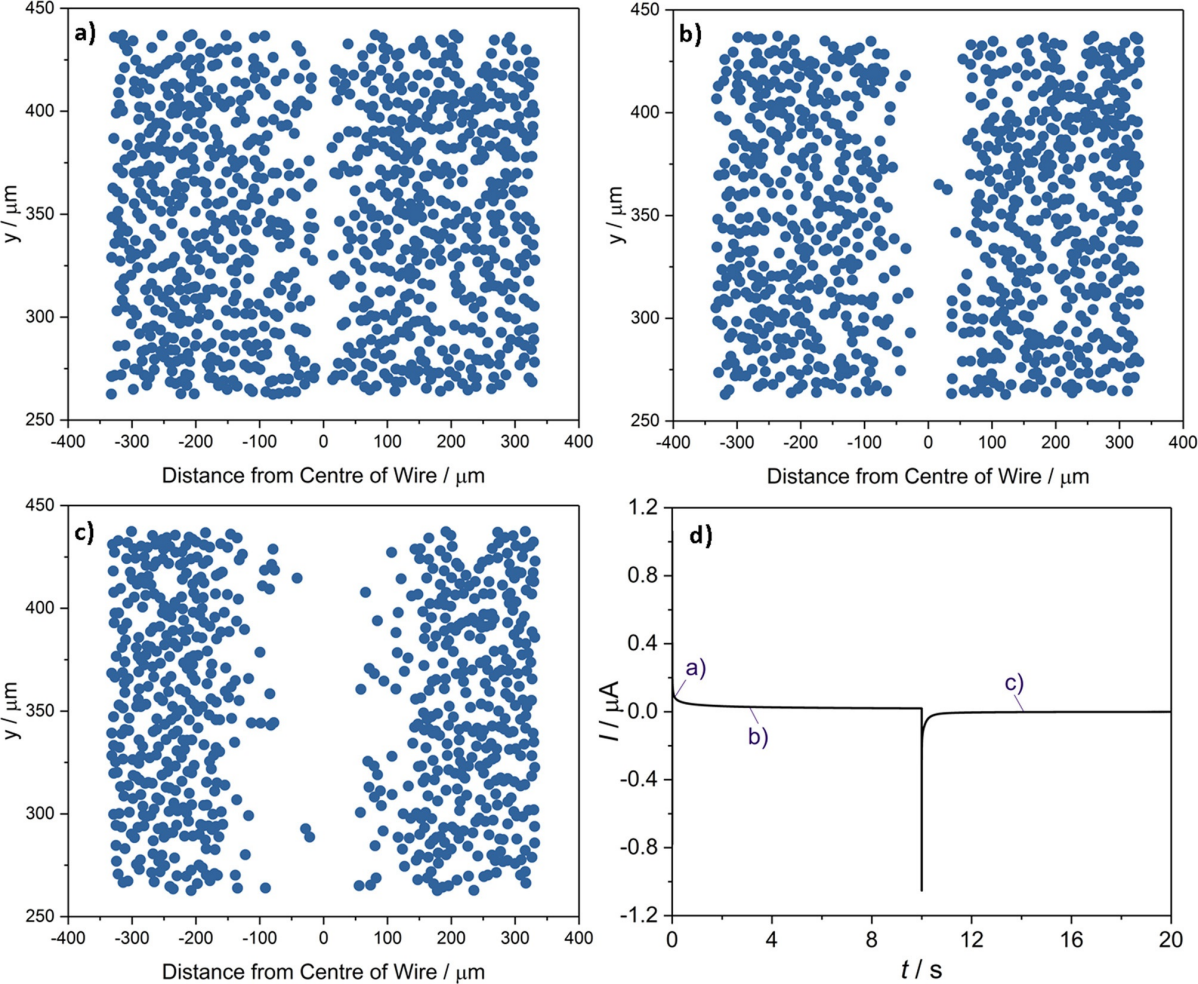
NP tracking images of a carbon fiber wire immersed in 1.2 pm AgNPs in 20 mm KBr as a function of time: a) 0 s, b) 3.1 s, c) 14.1 s. d) The accompanying double potential step chronoamperogram, in which the potential was held at 1.3 V (vs. pseudo‐Ag) for 10 s, before being stepped to 0.0 V for 30 s.

Double potential step chronoamperograms of suspensions of 1.2 pm AgNPs in 20 mm KBr were obtained at the supported carbon fiber wire, whilst the microscope concomitantly captured dark‐field images of the process at a rate of 10 fps. The potential was initially held at 1.3 V (vs. pseudo‐Ag) for 10 s before being stepped to 0.0 V for 30 s; Figure 4 d) depicts the chronoamperogram for the process; this time–current profile predominantly shows the current associated with capacitive discharging and charging of the electrode. During the course of this experiment, the concentration of the NPs within approximately 100 μm of the electrode becomes depleted, as evidenced optically. To facilitate the analysis and visualization of this result, the solution‐phase‐based NPs were identified in each frame. The raw dark‐field microscopy images from this experiment are presented in Figure 3. The method used in identifying the positions of the particles is outlined in the Experimental Section and representative results are presented in Figure 4 a–c, from three times during the course of the experiment. The AgNP concentration present in Figure 4 a can be estimated by taking the number of particles identified and dividing through by *N*_A_ and the volume of the cell. The numerical aperture of the objective used (0.25) affords a focal depth of 4 μm. Multiplying this by the area studied in Figure 4 gives a volume of 4.47×10^−13^ m^3^, and an average AgNP concentration of 3.7 pm, in good agreement with the expected value.

The depletion and exclusion of the NPs in the vicinity of the electrode can be clearly visualized. This depletion is not attributed to the direct oxidation of the NPs at the electrode surface, given that the length scale of the exclusion zone adjacent to the electrode is significantly greater than the predicted diffusion layer thickness for the AgNPs over this time frame [(2*Dt*)^0^⋅^5^ ≈20 μm for *t* ≈20 s]. Moreover, video evidence clearly demonstrates that this local exclusion is not caused by particle transport away from the electrode; the scattering intensity from individual NPs rapidly decreases during the course of the experiment. This evidence is presented in the Section S7 of the Supporting Information. Consequently, the origin of the NP exclusion zone is concluded to relate to the reaction of the NPs with a species formed at the electrode surface. This reaction leads to the formation of a reaction layer adjacent to the electrode surface; the length scale of this reaction layer is related to the rate of diffusion of the formed molecular species and not the mass transport of the particles themselves. The decrease in scattering from the NPs may arise from the redox‐driven dissolution of the AgNPs or their conversion into a less highly scattering (non‐plasmonic) material. These optoelectrochemical results are consistent with the oxidation of AgNPs in the diffusion layer of the electrode, as driven by a molecular oxidation process occurring at the electrochemical interface.

Figure 4 d shows that during the first 10 s at +1.3 V versus Ag the pseudo‐steady‐state current is approximately 20 nA. Given the known geometry of the cylindrical surface‐mounted electrode (length=1.5 mm, diameter=7 μm), this corresponds to a current density of 60 μA cm^−2^. Assuming the oxidation process corresponds solely to the oxidation of bromide and through simulation of the diffusion‐limited current for the one‐electron oxidation of bromide at the electrode, the mass‐transport‐limited flux of bromide to the electrode surface can be estimated to yield a current density of 13 mA cm^−2^. This approximate current value has been determined from consideration of the flux to an isolated cylinder and dividing by a factor of 2 to allow for the diffusional blocking of the glass substrate.^35^ The details of the simulation and the corresponding voltammogram are presented in Section S8 of the Supporting Information. Direct comparison of the theoretically determined mass‐transport‐limited flux to the experimental value of 60 μA cm^−2^ indicates that approximately 0.5 % of the bromide is being oxidized at the electrode surface.

Similar results to the above are obtained when the optical response of the system is studied using cyclic voltammetry, as compared to the double step chronoamperograms outlined above. To further verify the action of bromide oxidation as a cause, the optoelectrochemical responses of 1.2 pm AgNPs in 20 mm KCl and KF were also studied. By using KF as the supporting electrolyte, cyclic voltammograms with first vertex potentials as high as 2.8 V returned no optical effect on the AgNPs in the vicinity of the electrode, namely, a reduction in NP concentration in a layer around the electrode is not observed.

However, it should be noted that, at these high potentials, small clusters of material began to accumulate at the wire electrode, potentially owing to polymerization of citrate at the surface. The absence of the response even at these very high potentials further confirms that the dissolution of AgNPs is not caused by products of solvent oxidation. With KCl as the supporting electrolyte, a similar but less defined reaction layer is observed. Here, a higher potential was required to initiate the dissolution of the AgNPs. This may be attributed to the inefficiency of the chlorine evolution reaction (CER)^32^ process leading to concurrent oxidation of the chloride and the solvent at higher potentials.

### 2.3 Theoretical Models of the NP Reaction layer

This section serves to outline theoretical models of the solution‐phase reaction between bromine and the AgNPs, leading to the dissolution of the particulate material in the vicinity of the electrode. The pertinent reactions are:

(1) $${Br}^{-}-e^{-}\to \;\frac{1}{2}{Br}_{2}$$

(2) $$AgNP+\frac{n_{Ag}}{2}{Br}_{2}\to n_{Ag}AgBr$$

where Reaction (1) is driven at the electrochemical interface and Reaction (2) occurs in the solution phase at distances away from the electrochemical interface. The product of bromide oxidation, as shown in Reaction (1), is taken to be bromine. However, the possibility of bromine speciation must also be considered. The equilibrium constant of the reaction of bromide and bromine to form tribromide in aqueous solution is extremely low (*K*_eq_=5.7×10^−9^ at 25 °C), and the reactions of bromine and water to form hypobromite and bromate are similarly disfavored.^36^ In this non‐buffered solution, it is consequently extremely likely that the overwhelming product of the reaction is indeed bromine, as described by Reaction (1). In Reaction (2), the bromine formed at the electrochemical interface may diffuse away from the surface and subsequently react with the AgNPs. Here, in Reaction (2), the value of *n*_Ag_ is approximately 2.2×10^6^ and represents the average number of silver atoms per AgNP. The experimental AgNP concentration is 1.2 pm; consequently, the solution contains in total only micromolar concentrations of silver atoms.

Also contained in the theoretical models, is the possibility for the NPs to be directly consumed at the electrode surface in accordance with the following reaction:

(3) $$AgNP-\;n_{Ag}e^{-}\to n_{Ag}{Ag}^{+}$$

The complexation of the formed argentous ions with the solution‐phase bromide is not accounted for in Reaction (3). However, owing to the markedly different diffusion coefficients associated with the bromine and the AgNPs of 1.0×10^−9^ and 1.0×10^−11^ m^2^ s^−1^, respectively, the approximation made by using Reaction (3) leads to no significant error, that is, the silver oxidation is dominated by its reaction with the formed bromine.

In the experimental cell, the cylindrical carbon fiber electrode is supported on the glass surface; this geometry may be appropriately approximated as a hemicylinder, thus reducing the coupled mass transport–chemical kinetics problem to one dimension. In the following models, only the direction perpendicular to the electrode surface radius *r* is accounted for. In the double potential step chronoamperometry experiment, Br^−^ is oxidized to Br_2_ at the first potential step *E*_1_ and Br_2_ is reduced to Br^−^ at the second potential step *E*_2_ [Eqs. (4), (5)]:

(4) $$c_{{Br}_{2}}\left(r=r_{el}\right)=const\times c_{{Br}^{-}}^{{}^{*}},\;c_{{Br}^{-}}\left(r=r_{el}\right)=\left(1-2\times const\right)c_{{Br}^{-}}^{{}^{*}}\;\;\;at\;E_{1}$$

(5) $$c_{{Br}_{2}}\left(r=r_{el}\right)=0,\;c_{{Br}^{-}}\left(r=r_{el}\right)=c_{{Br}^{-}}^{{}^{*}}\;\;\;at\;E_{2}$$

where $c_{{Br}^{-}}^{*}$ is the bulk concentration (*r* → ∞ ) of Br^−^ and *c*(*r*=*r*_el_) refers to the surface concentration at the hemicylinder electrode with the radius of *r*_el_. Before the redox reaction, there is only Br^−^ in solution and the surface concentration of Br^−^ equals $c_{{Br}^{-}}^{*}$ . After applying a potential, the surface concentration varies as a function of potential. At the first potential step *E*_1_ in the double potential step chronoamperometry, a small portion of Br^−^ is oxidized to be Br_2_. In Equation (4), *const*=0.005 is a fitting parameter, which is not calculated from any assumption of the electron‐transfer kinetics, but directly determined by fitting to the current of the first potential step in Figure 4 d. At the second potential *E*_2_, Br_2_ is fully reduced to Br^−^ at the electrode and the surface concentration of Br_2_ is therefore zero. At both electrode potentials, the AgNPs can be oxidized at the electrode surface [Eq. (6)]:

(6) $$c_{AgNP}\left(r=r_{el}\right)=0$$

AgNPs in the solution can be also oxidized by Br_2_ produced via the redox reaction during the first potential step.

**Figure 5:**
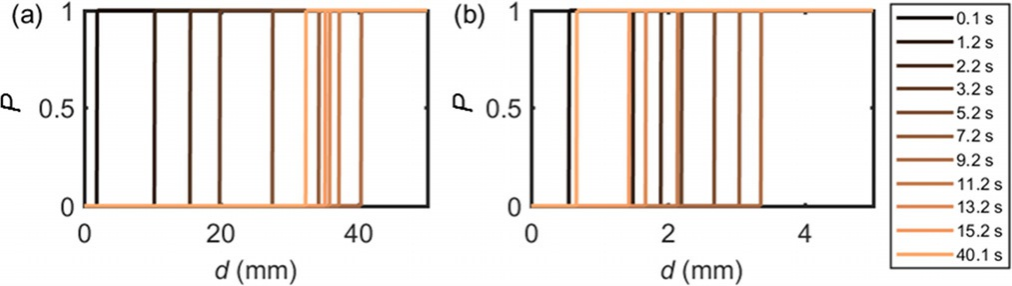
Simulated concentration profiles with various reaction times in the double potential step chronoamperometry. a) The maximum amount of AgNPs consumed by all the Br_2_ generated at the electrode; b) the case where only the mass transport of Br_2_ is accounted for.

Three models are proposed to understand the experimentally formed NP reaction layer. In the first and simplest model, the mass transport of bromine is not considered, and it is assumed that all bromine formed immediately reacts with the AgNPs. The amount of Br_2_ generated during the double potential chronoamperogram *N* ${}_{\mathrm{Br}{}_{2}}$ (mol) can be calculated by the charge integrated from the electrode current *I* (A) over the reaction time *t* (s) [Eq. (7)]:

(7) $$N_{{Br}_{2}}=\frac{\int Idt}{2F}$$

where *F*=96 485 C mol^−1^ is the Faraday constant. Assuming the concentration of AgNP in solution $c_{AgNP}$ remains constant, the amount of AgNP *N*_AgNP_ at distance *d* from the microwire electrode center can be calculated [Eq. (8)]:

(8) $$N_{AgNP}=\;2\pi dl_{el}c_{AgNP}$$

where *l*_el_ is the length of the microwire electrode. As the number of silver atoms in one AgNP *n*_Ag_ is approximately 2.2×10^6^ on average, there is approximately 1.1×10^6^ Br_2_ needed to oxidize one AgNP. If the mass transport of both the AgNPs and Br_2_ is ignored and assuming all Br_2_ generated from the electrode reacts with AgNPs, the “titrated” concentration profiles of AgNPs at various reaction times can be constructed, as shown in Figure 5 a. In Figure 5 a, the region of *P*=0 is the reaction layer, where $N_{AgNP}<\frac{n_{Ag}}{2}N_{{Br}_{2}}$ , reflecting that AgNPs are removed by Br_2_. *P* in Figure 5 represents the probability of observing AgNPs in these models. The thickness of the AgNP reaction layer increases over the first 10 s and then decreases for the rest of the process, as a result of Br_2_ first being produced and then reduced at the electrode surface during the double potential step chronoamperometry. From Figure 5 a, it is immediately clear that the reaction layer length scale predicted by the model is not consistent with experiment, where only NPs within 350 μm of the electrode are seen to be influenced by the electrochemical reaction. This situation partially reflects the fact that, experimentally significantly, more bromine is formed at the electrode (ca. 3×10^−14^ moles of Br_2_ formed compared to ca. 1×10^−20^ moles of AgNPs); but, over the course of the experiment, only a relatively small fraction of this material reacts with the AgNPs.

In the second model, the diffusion of bromine is accounted for and depicted by Fick's second law [Eq. (9)]:

(9) $$\frac{\partial c_{{Br}_{2}}}{\partial t}={D_{{Br}_{2}}\nabla }^{2}c_{{Br}_{2}}=D_{{Br}_{2}}\left(\frac{\partial^{2}}{\partial r^{2}}+\frac{1}{r}\frac{\partial }{\partial r}\right)c_{{Br}_{2}}$$

Solving Equation (9) with boundary conditions (4) and (5), the concentration of Br_2_ can be calculated as a function of both reaction time and the distance from the electrode. The concentration of *c*_AgNP_ is still treated as constant in this model. Similarly, we define *P*=0 as the region where $c_{AgNP}<\frac{n_{Ag}}{2}c_{{Br}_{2}}$ . Figure 5 b depicts this simplified model, where the mass transport of Br_2_ is simulated but the influence of the solution‐phase reaction kinetics on the local Br_2_ concentration is not accounted for. In comparison with the idealized “titration” model presented in Figure 5 a, and accounting for the mass transport of Br_2_, the length scale of the reaction layer of AgNP depicted in Figure 5 b is significantly contracted. This highlights the important role the bromine mass transport plays in controlling the reaction layer length scale. However, the experimentally determined reaction layers are still found to be significantly smaller and finite compared to that predicted by this model, indicating the likely importance of the finite kinetics of the reaction between the bromine and AgNPs.

**Figure 6:**
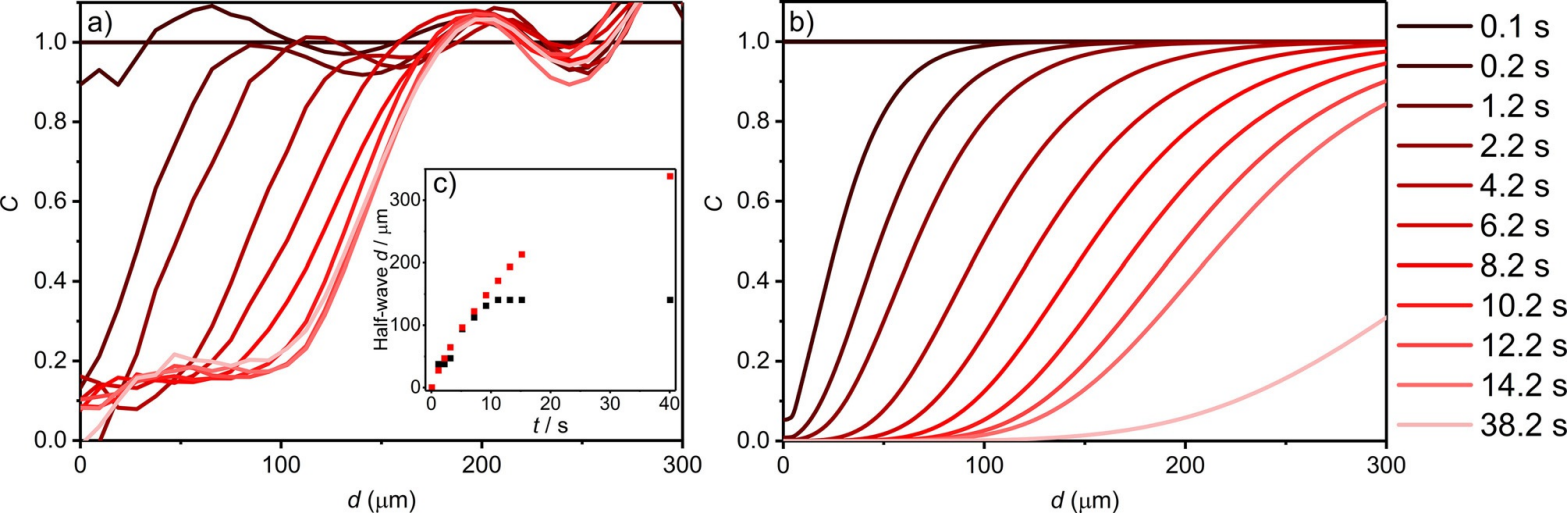
a) Experimental and b) simulated NP concentration profiles for a suspension of 1.2 pm AgNPs in 20 mm KBr as a function of time, the simulation assuming a rate constant *k*_sol_=30 mm ^−1^ s^−1^. c) Half‐wave (*C*_AgNP_=0.5) distances for the experimental (black squares) and simulated (red squares) concentration profile as a function of time.

In the third model, which is applied to simulate the experimental data in Figure 6, the reaction is simplified and modelled as an irreversible second‐order homogeneous chemical reaction with a rate constant *k*_sol_ (mm ^−1^ s^−1^). The mass transport of AgNPs and Br_2_ is described by the diffusion equation, Fick's second law, combined with the chemical reaction between the AgNPs and Br_2_. The chemical reaction is approximated to be a second‐order reaction [Eqs. (10), (11)]:

(10) $$\frac{\partial c_{{Br}_{2}}}{\partial t}=D_{{Br}_{2}}\nabla^{2}c_{{Br}_{2}}-\frac{n_{Ag}}{2}k_{sol}c_{{Br}_{2}}c_{AgNP}$$

(11) $$\frac{\partial c_{AgNP}}{\partial t}={D_{AgNP}\nabla }^{2}c_{AgNP}-k_{sol}c_{{Br}_{2}}c_{AgNP}$$

Further details on the modelling of the electrode system is presented in Section S9 of the Supporting Information. Compared to the first and second models in Figure 5, the third model provides a better fitting to the experimental data, which is shown in Figure 6 and will be discussed in the next section.

Further consideration must be given to the possibility of silver bromide reacting to form higher halides (AgBr_2_^−^ and AgBr_3_^2−^ are known to exist).^37^, ^38^, ^39^ Alternatively, the product of the reaction could be solid silver bromide; considering its sparing solubility (*K*_sp_=7.7×10^−13^ at 298 K)^40^ and with the small amount formed, it would likely be extremely dilute. For the purposes of the simulation, by modelling the reaction as an irreversible process, any secondary reaction or formation of higher halides can be neglected; the reaction consumes bromide, not bromine. This can be rationalized by considering the electrode potentials of Br_2_ to Br^−^ and Ag to Ag^+^ (+1.07 V and +0.80 V vs. SHE respectively).^31^ This reaction is clearly driven, hence, is likely irreversible. The *k*_sol_ term should be dependent on the surface area to volume ratio of the NP. The rate‐determining step of the process may be either this surface reaction, or the mass transport of the bromine into the core of the silver. A further limitation of this approach is the assumption that the *k*_sol_ is constant. The theoretical models here have served to outline the basis of the solution‐phase reaction between bromine and the AgNPs. The developing of the first two molecular based models to the third NP‐based model allows for a clear comparison with the experimentally obtained data, as is presented in the next section.

### 2.4 Comparison of Experimental and Theoretical Data

The NP positions identified in the microscope images, as presented in Figure 6 a–c, enable the relative local NP concentration to be quantified during the course of the electrochemical reaction. The NP identification data extracted from the microscope images were enumerated by calculating the local particle concentrations as a function of distance from the center of the wire, the results of which are depicted in Figure 6 a. The process employed is detailed in the Experimental Section and provides a clear measure of the distance over which the NPs are oxidized in the vicinity of the electrode.

Figure 6 depicts the local NP concentration profiles over the timescale of the experiment, compared with those from the simulated model, assuming a rate constant of *k*_sol_=30 mm ^−1^ s^−1^. Fitting of the data with rate constants of 10 and 50 mm ^−1^ s^−1^ is presented in Section S10 a of the Supporting Information. Figure 6 c (inlay) presents the half‐wave (*C*_AgNP_=0.5) distance comparison for experiment and simulation. The experimental and simulated half‐wave distances show excellent correlation for6s<t<10s . The reduction in concentration of the AgNPs as the bromine diffuses away from the electrode is clearly evident.

We note that, although the simulated concentration profiles are generally comparable with the observed data, there is an apparent discrepancy between the two profiles at 0.2 s. This can be attributed to the poor resolution of the NPs at distances very close to the interface. It was noted that above that, owing to the intense scattering from the carbon fiber electrode, no NPs can be resolved around 13.5 μm either side of the wire, meaning any NPs 10 μm from the edges of the wire are not imaged. The fluctuations above *C*_AgNP_=1 in Figure 6 a are an artefact of the smoothing filter applied to the data, as detailed in the Experimental Section. Moreover, as depicted in Figure 6 c, the simulation predicts that far more bromine is made than is consumed, as also reflected in the models presented in Figure 5, and that this layer of bromine should continue to move outwards long after bromine formation concludes. However, we observe experimentally that the layer stops expanding after approximately 14 s. This lack of correspondence between simulation and theory at longer times likely arises from one or more of the approximations used in the simulation. This may be attributed to the simplification of the reaction as an *irreversible* second‐order reaction with a *constant* rate constant *k*_sol,_ it is likely that this value changes over the course of the reaction. Moreover, the simulation assumes the mass transport to be a diffusion‐only process; at these large timescales (>10 s), one may anticipate convection within the cell to become an important factor.^41^ However, it should be noted that no drift was immediately apparent in the optical microscope images, and such convective flows in the *z* direction would likely lead to flows in the *x* and *y* directions of the microscope images.

The kinetics of the AgNP bromine reaction clearly control the length scale of the reaction layer, compressing it to lengths of only a few hundred microns. The combined opto‐electrochemical cell has enabled the visualisation of the solution phase reaction and demonstrated that the inhibition of the NP impacts is as a result of their reaction with the electrode product in the vicinity of the electrode.

## 3 Conclusions

From an environmental and biological standpoint, the study of the redox chemistry of AgNPs in the solution phase and not at a conductive surface is important for understanding their fate and influence in larger systems. These NPs can be described as isolated microelectrodes in solution, sensitive to the local electrochemical environment. As such, dark‐field microscopy can be used to dynamically track NPs in the solution phase. This work has first served to demonstrate how the electrochemical detection of AgNPs can be inhibited at an electrode surface at high electrode potentials. With the use of a combined optoelectrochemical cell, it was demonstrated that this inhibition occurs because the particles in the electrode diffusion layer react with the product of oxidation at the electrode. Modelling and analysis of the optical results demonstrate and provide physical insight into the importance of the solution‐phase reaction kinetics in controlling the extent of this local particle depletion. The complexities associated with the modelling of this solution‐phase NP reaction are duly highlighted. The local particle depletion observed is concluded to relate to the reaction of the AgNPs with the product of oxidation, bromine, at the electrode as it diffuses away. It has been demonstrated that the reaction of AgNPs with bromine in the solution phase is a kinetically limited process and that the scale of the reaction layer is dependent on the rate of diffusion of bromine, as opposed to the mass transport of the particles themselves.

The new methods developed offer a route by which the redox chemistry of AgNPs may be studied in the solution phase without recourse to ensemble techniques such as UV/Vis spectroscopy. Furthermore, the methodology developed in this work may be extended to probe the processes occurring in reactions of other highly scattering electroactive materials.

## Experimental Section

### Chemical Reagents

Commercial spherical citrate‐AgNPs of 50 nm diameter (NanoXact, 0.02 mg mL^−1^ silver, 2 mm sodium citrate) were obtained from Nanocomposix, USA. Representative TEM images of the NPs are presented in Figure S1. All other reagents were purchased from Sigma–Aldrich and were used as received, without further purification: potassium fluoride (≥99.0 %), potassium chloride (≥99.0 %) and potassium bromide (≥99.0 %). All solutions were prepared by using deionized water (Millipore) with a resistivity of no less than 18.2 MΩ cm at 25 °C.

### Stability of the AgNPs in KBr

The stability of AgNPs in KBr (20 mm) and deionized water was studied by using UV/Vis measurements (*λ*=250–700 nm) with a Shimadzu UV‐1800 spectrophotometer in disposable cuvettes (Eppendorf UVette, Sigma–Aldrich) with a 10 mm optical path length. UV/Vis spectra were recorded over a 90 min period after potassium bromide was added to form a suspension of AgNPs (1.2 pm) in KBr (20 mm). Over this 90 min period, the peak absorbance in KBr drops only 3.4 % from its initial value, with the magnitude of the peak absorbance (*λ*_max_=425±1 nm) remaining constant for the suspension in water. The resulting spectra and subsequent discussion are presented in Figure S2.

### Nanoimpacts

Nanoimpact experiments were performed by using a home‐built, low‐noise potentiostat as described previously.^42^ The low‐noise current amplifier (LCA‐4K‐1G, FEMTO Messtechnik GmbH, Germany) was filtered by using two cascade analog RC‐filters at 2k Hz, the signal was subsequently digitized at 100 KS s^−1^ via a USB data acquisition device (USB‐6003, National Instruments, Texas, US). Finally, the signal was filtered digitally (4‐pole Bessel) to 100 Hz by using a script written in Python 3.5. Measurements were made at a carbon microdisc (33 μm diameter, IJ Cambria Scientific Ltd, UK). A leakless Ag/AgCl (in 3.4 m KCl, eDAQ) or a saturated calomel electrode (SCE; BASi, USA) was used as a reference, and a platinum wire was used as a counter electrode. Nanoimpacts performed in KF and KBr were performed against the leakless Ag/AgCl reference electrode (*E*=−0.039 V vs. SCE) to prevent chloride contamination, but all results are reported against a SCE reference electrode for clarity.

Cyclic voltammetric measurements were carried out on AgNP (12 pm) solutions in different supporting electrolytes (KF, KCl, KBr, all 20 mm), scanning anodically in the potential range of 0.0 to 1.4 V for KF and KCl, and −0.1 to 1.0 V for KBr at a scan rate of 10 mV s^−1^. This was performed by using the carbon microdisc electrode. Prior to use and between scans, the working microelectrode was polished on aqueous slurries of 1.0, 0.3, and 0.05 μm alumina in descending order of size, before the alumina residue was removed by rinsing with deionized water. Measurements were repeated with four consecutive scans for the suspensions in KBr (20 mm), scanning anodically in the potential range of 0.15 to 0.3 V. Further measurements were taken on the KBr system, varying the immersion time of the electrode prior to the scan from 10 to 60 s.

### Optical Setup and Optoelectrochemical Cell Design

Optical measurements were made on a Zeiss Axio Examiner.A1 microscope by using a 20× air objective (NA=0.5, EC Plan‐Neofluar), and an ultra‐dark‐field 1.2/1.4 oil condenser (both Carl Zeiss Ltd., Cambridge, UK). Image acquisition was provided by a Hamamatsu ORCA‐Flash 4.0 Digital CMOS camera (Hamamatsu, Japan), providing 16 bit images with 4 megapixel resolution. Images were acquired with an exposure time of 30 ms and at a rate of 10 fps.

The home‐built optoelectrochemical cell used consisted of a three‐electrode setup, wherein two 7.0 μm diameter carbon fiber wires (Goodfellow Cambridge Ltd., UK) acted as the working and counter electrodes and a third carbon fiber wire coated with a thin layer of silver epoxy (RS Components Ltd., UK) was used as a pseudo‐reference electrode. A schematic of the cell setup used is depicted in Section S3 of the Supporting Information. The glass slides and cover slips used were cleaned in Aqua Regia for at least 16 h and were then washed thoroughly with deionized water and acetone, prior to cell fabrication. The solution was transferred to the cell before a cover slip was placed on top to seal it. Potentiostatic control and synchronization with the camera were provided by an in‐house device, as described previously.^43^

### Optoelectrical Measurements and Analysis

Combined optoelectrochemical measurements were performed by using the setup described above. Initially, cyclic voltammograms were recorded in solutions containing AgNPs (1.2 pm) in KBr (20 mm), scanning first anodically in the potential range of 0.0 to 1.4 V (vs. pseudo‐Ag) at a scan rate of 50 mV s^−1^. This was repeated using KCl or KF (both 20 mm) as the supporting electrolyte. Then, double potential step chronoamperometry was carried out on solutions containing AgNPs (1.2 pm) in KBr (20 mm). The potential was initially held at 1.3 V (vs. SCE) for 10 s, before being stepped to 0 V for 30 s. Image processing and intensity analysis were performed in Zen 2 pro (Carl Zeiss Ltd., Cambridge, UK). For the chronoamperograms, NP identification was enabled via the use of Python script written in Python 3.5. The script identified the NPs in a given frame according to a set of user‐defined parameters. Having identified the NP positions in the microscope image, the localized concentration profiles could be readily determined. The *y* coordinates of all particles in the defined range *x=*750 to 1250 px (262.5 to 437.5 μm) were returned and binned for each frame. Smoothing of the binned data was achieved by using a twelfth‐order Savitsky–Golay^44^ filter, points of window size=46. The data was then normalized with respect to the first frame, and averaged over periods of 10 frames (1 s), before being averaged symmetrically about the center of the wire.

## Conflict of interest

*The authors declare no conflict of interest*.

## Supporting information

As a service to our authors and readers, this journal provides supporting information supplied by the authors. Such materials are peer reviewed and may be re‐organized for online delivery, but are not copy‐edited or typeset. Technical support issues arising from supporting information (other than missing files) should be addressed to the authors.

Supplementary

Supplementary
