# Supplementary material for: Coupled Optical and Electrochemical Probing of Silver Nanoparticle Destruction in a Reaction Layer
Source: ChemistryOpen. 2018 May 23;7(5):370–80. doi: 10.1002/open.201800048 (PMC5974555; doi:10.1002/open.201800048)
Supplement: Supplementary file 1 — Supplementary [file OPEN-7-370-s001.pdf]

ChemistryOPEN

Supporting Information

© 2018 The Authors. Published by Wiley-VCH Verlag GmbH & Co. KGaA, Weinheim

Coupled Optical and Electrochemical Probing of Silver Nanoparticle Destruction in a Reaction Layer

Christopher A. Little, Christopher Batchelor-McAuley, Kamonwad Ngamchuea, Chuhong Lin, Neil P. Young, and Richard G. Compton*[a]

open_201800048_sm_miscellaneous_information.pdf

Contents

S1: TEM Images of AgNPs S-2

S2: UV/Vis Spectroscopic Study into the Stability of AgNPs in KBr S-3

S3: Optoelectrochemical Cell Design S-4

S4: Effect of Immersion Time on the Anodic Stripping Wave S-5

S5: Multi-Scan Cyclic Voltammograms of AgNPs in KBr S-6

S6: Charge Distribution of AgNPs S-7

S7: Video Evidence of Reaction Layer Formation S-8

S8: Simulated Voltammetric Response for an Irreversible System S-9

S9: Simulation of Double Potential Step Chronoamperometry S-10

S10: Concentration Profiles from Simulated Data S-12

References S-14

S1: TEM Images of AgNPs

The AgNPs were imaged using transmission electron microscopy (TEM) on a JEOL JEM-3000F instrument using an accelerating voltage of 300 kV. Sample preparation was carried out by drop-casting suspensions of nanoparticles onto carbon grids (Agar Scientific) and allowing these grids to dry. The images were extracted using ImageJ software.

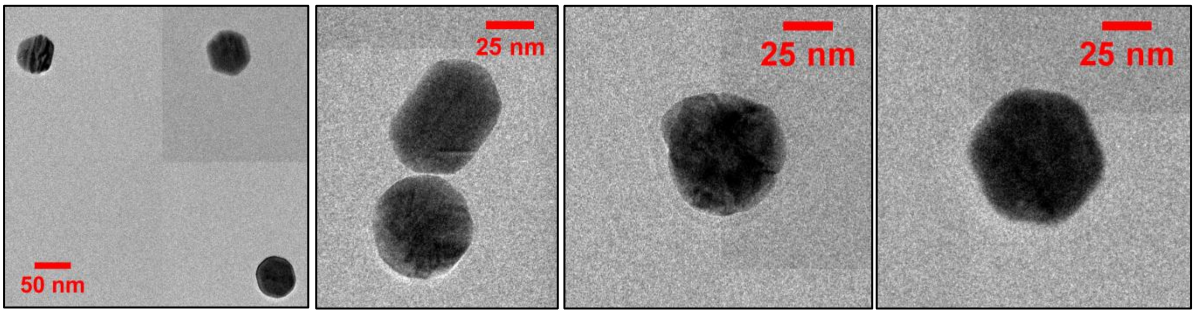

Figure S1. Representative TEM images of commercial spherical citrate-AgNPs of 50 nm diameter (NanoXact, 0.02 mg mL^{-1} silver, 2 mM sodium citrate)

S2: UV/Vis, Spectroscopic Study into the Stability of AgNPs in KBr

Whilst AgNP suspensions exhibit a strong plasmonic peak at around 400 nm, the magnitude, shape and position of the peak are dependent on the size, geometry, and local environment of the AgNPs.[1] The magnitude for the maximum absorbance can be used to assess the stability of the suspensions over time. Figure S2 presents the UV-Vis spectra of 1.2 pM AgNPs in 20 mM KBr as a function of time, with the equivalent spectrum in deionized water overlaid. The magnitude of the peak absorbance of the AgNPs over a 90 minute period is inlaid. Over this 90 minute period, the peak absorbance in KBr drops only 3.4% from its initial value, with the magnitude of the peak absorbance (λ_{max} = 425 ± 1 nm) remaining constant for the suspension in water. This demonstrates that over the time period of the cyclic voltammograms and chronoamperograms being run, the AgNPs particles are suitably stable under such conditions.

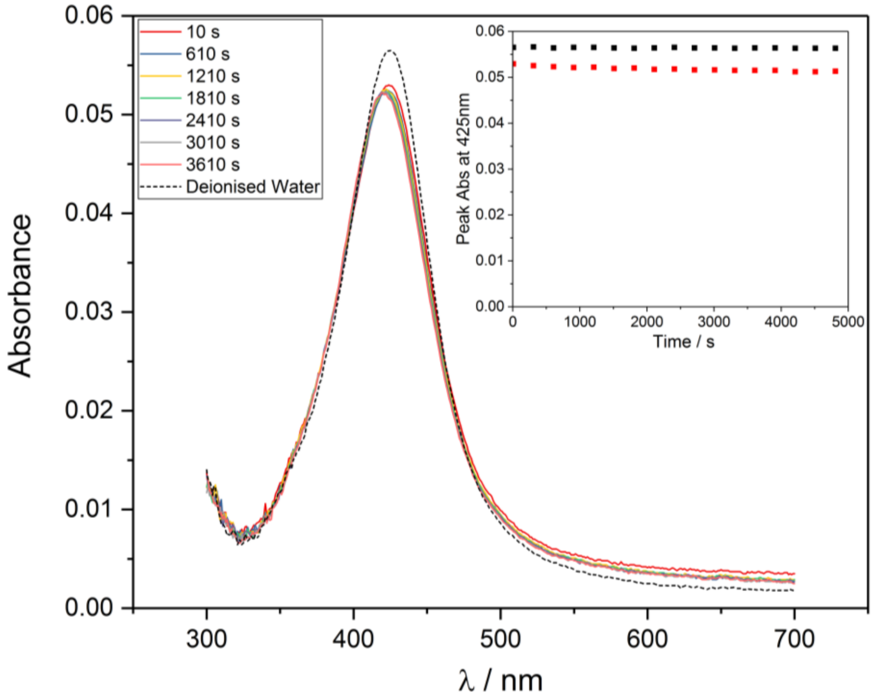

Figure S2. UV-Vis spectra of 1.2 pM AgNPs in 20 mM KBr over 10 minute periods. The spectrum in deionised water is overlaid for comparison. The inlay depicts the peak absorbance at 425 nm for AgNPs in deionized water (black squares) and in 20 mM KBr (red squares) over a period of 90 minutes.

S3: Optoelectrochemical Cell Design

A schematic of the thin-layer cell manufactured for use in opto-electrochemical measurements is depicted in Figure S3. Carbon Fibre wire (7 μm diameter) was attached to a conducting wire (0.2 mm diameter) of around 5 cm in length using the electrically conducting silver loaded epoxy adhesive (RS, USA) to facilitate electrochemical measurements. The wires were sealed to the surface with a layer of electrically insulating Kapton tape, with the walls of the cell derived from 6 layers of this tape. "Nail polish" consisting of nitrocellulose in organic solvents acts as a hydrophobic barrier to seal the Kapton tape and wire to the surface, and to prevent any leakage into the electrical connections.

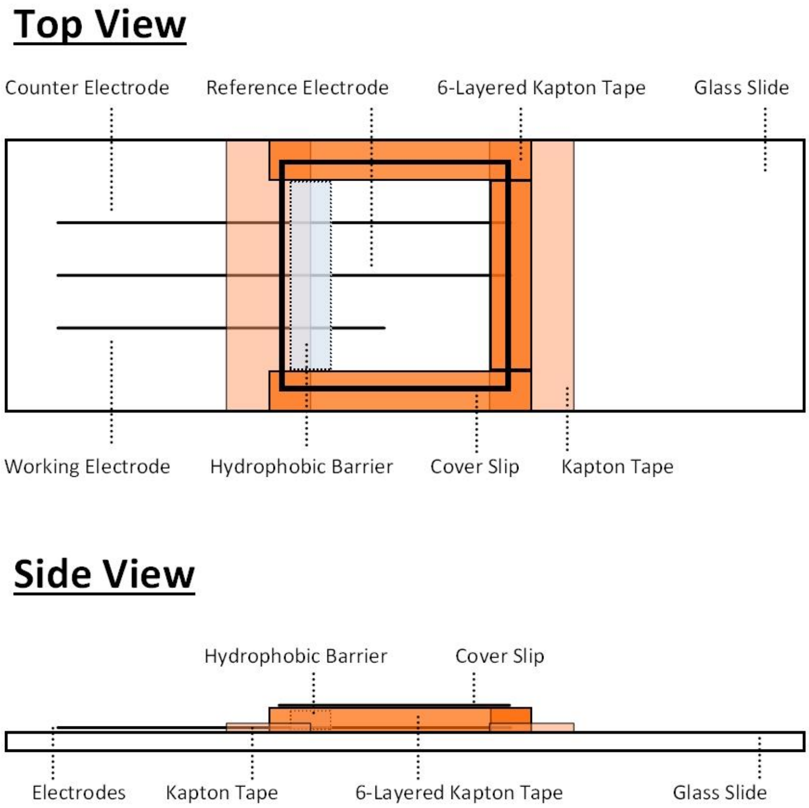

Figure S3. Schematic of optoelectrochemical cell used in concomitant double step chronoamperometry and dark-field imaging.

S4: Effect of Immersion Time on the Anodic Stripping Wave

Figure S4 depicts the change in the magnitude of the anodic stripping wave as a function of the electrode immersion time prior to the scan. The magnitude can clearly be seen to grow as a function of time, evidencing a larger number of AgNPs that have been pre-adsorbed to the electrode being oxidised. By taking the mean charge of an AgNP to be 0.4 pC and the steady state flux to the electrode to be 4.7 Hz (see main text), the red squares in the inlay depict the expected total charge under the stripping wave as a function of immersion time, compared to the experimentally obtained values. The experimentally determined values are calculated by integrating the area under the anodic stripping wave relative to the baseline of signal, and dividing through by the scan rate (10 mV s^{-1}) to obtain the charge. Having neglected the process of placing the electrode into the solution and the subsequent convection, these are comparable within an order of magnitude and suggest that the pre-adsorption onto the electrode is irreversible.

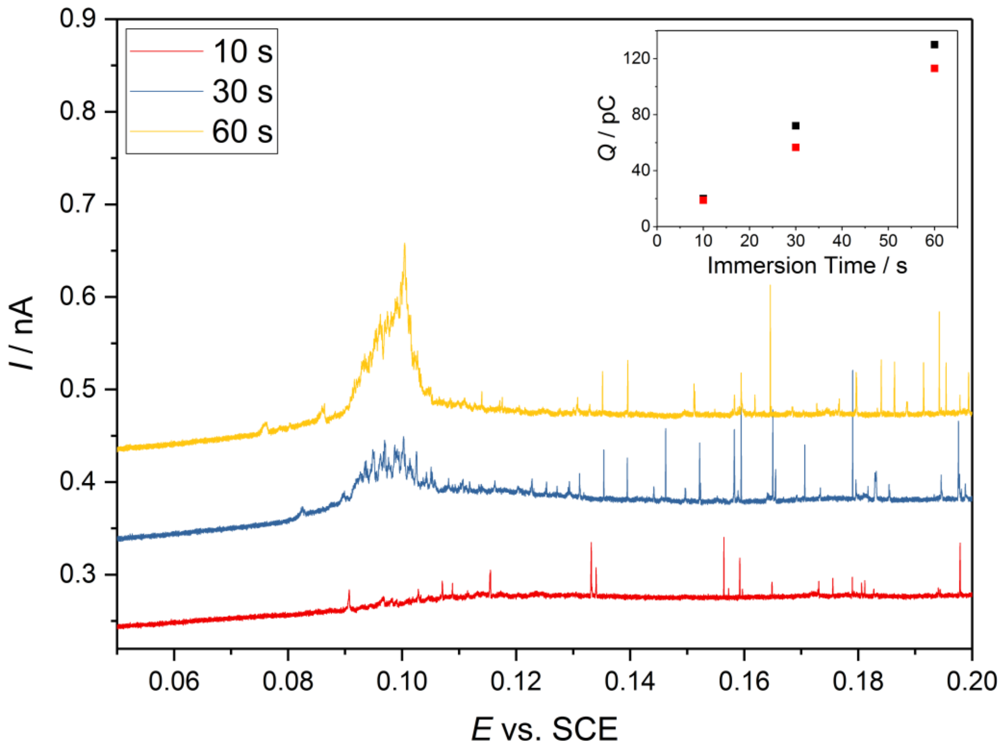

Figure S4. Representative voltammograms of 12 pM AgNPs in 20 mM KBr with immersion times of 10 s (red line), 30 s (blue line) and 60 s (yellow line) before scanning is initiated. Inlay presents the expected charge under the stripping wave (red) compared to the experimentally observed value (black).

S5: Multi-Scan Cyclic Voltammograms of AgNPs in KBr

Four consecutive cyclic voltammetric scans were run on a solution of 12 pM AgNPs in 20 mM KBr starting at a potential above the threshold at which oxidative spikes are observed to determine if the same reduction in spikes is observed with a lower first vertex potential. Discarding the first scan due to the effect of the irreversible adsorption of nanoparticles at the electrode, the cumulative number of spikes observed as a function of time for the three remaining scans is presented in Figure S5. It is evident that the gradient of the line is independent of the direction of the scan, and the cumulative number of spikes agrees well with the theoretically predicted value assuming a steady state flux to the electrode.

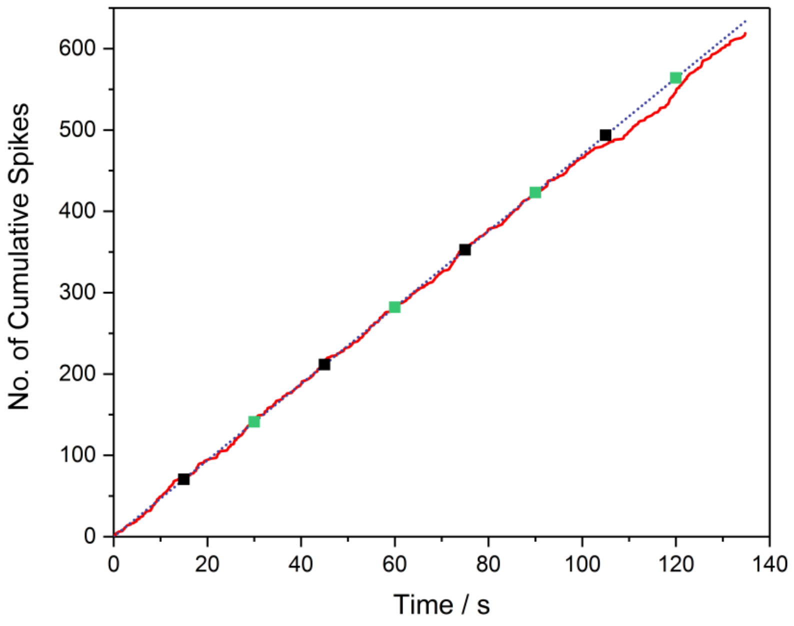

Figure S5. Cumulative number of spikes recorded as a function of time with 12 pM AgNPs in 20 mM KBr (red line). The data originates from 4 consecutive scans from 0.15 V to 0.30 V at a scan rate of 10 mV s^{-1}, with the first scan discarded due to the accumulation of nanoparticles on the electrode. Vertex potentials of 0.3 V and 0.15 V are displayed as green and black squares respectively. The purple dashed line depicts the expected cumulative number of spikes assuming a steady-state flux to the electrode.

S6: Charge Distribution of AgNPs

Figure S6 presents the charge distributions extracted by integrating under the oxidative spikes present in the six cyclic voltammograms of the suspensions of 12 pM AgNPs in 20 mM KBr. The spikes on the forward and backward scans are analysed separately, and depicted as cumulative frequency plots for clear comparison. The 2426 total spikes on the forward scans and 755 total spikes on the backwards scans were found to have means of 0.402 ± 0.006 pC and 0.274 ± 0.007 pC respectively. The cumulative frequency plots clearly demonstrate a statistically significant increase in the proportion of smaller spikes during the backward scans. This supports the notion that the smaller, faster moving AgNPs reach the electrode faster than their larger counterparts following the clearing out of AgNPs at high potentials.

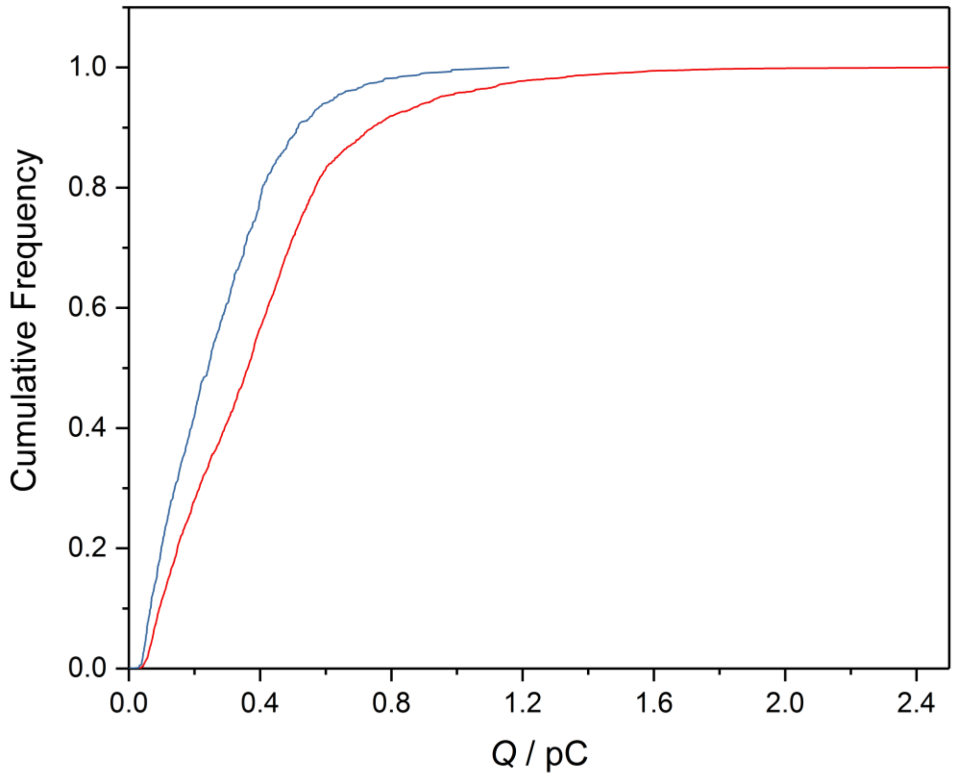

Figure S6. Cumulative frequency plots of the charge transferred during nano-impacts of 12 pM AgNPs in 20 mM KBr, split by forward scan (red) and backward scan (blue).

S7: Video Evidence of Reaction Layer Formation

AgNPs_Dissolution.avi

Double potential step chronoamperograms of suspensions of 1.2 pM AgNPs in 20 mM KBr were performed at a supported carbon fibre wire (7 μm diameter) whilst the microscope concomitantly captured dark field images of the process at a rate of 10 fps. The potential was initially held at 1.3 V (vs. pseudo-Ag) for 10 s before being stepped to 0.0 V for 30 s. The video, available online, presents the first 10 seconds of this chronoamperogram at 10 fps over the area of the image analysed using particle tracking software. The video clearly demonstrates that the local exclusion of nanoparticles is not due to mass transport away from the electrode; the scattering intensity from individual nanoparticles can instead be seen to decrease with time.

S8: Simulated Voltammetric Response for an Irreversible System

The commercial package DigiSim was used to simulate the voltammetric response for the oxidation of bromide to bromine, in order to determine the diffusion limited current for the system, assuming irreversibility. The parameters used were detailed as follows:[2-3]

$E_f^0 = 0.3\ \mathrm{V},\ k^0 = 1 \times 10^{-8}\ \mathrm{cm\ s^{-1}},\ \nu = 50\ \mathrm{mV\ s^{-1}},\ \alpha = 0.5$

$D_{Br^-} = 1.87 \times 10^{-5}\ \mathrm{cm^2\ s^{-1}},\ D_{Br_2} = 1.18 \times 10^{-5}\ \mathrm{cm^2\ s^{-1}}$

A cylindrical geometry was used assuming a radius and length of 3 μm and 1.5 mm respectively. The resulting voltammogram is presented in Figure S8. As referenced in the main text, the supported cylinder used in the opto-chemical response requires a correction factor of around 0.5 to approximate the change in geometry. This results in a diffusion limited current of ca. 4.4 μA, corresponding to a current density of 13.3 mA cm^{-2}, compared to 60 μA cm^{-2} in the observed experiment.

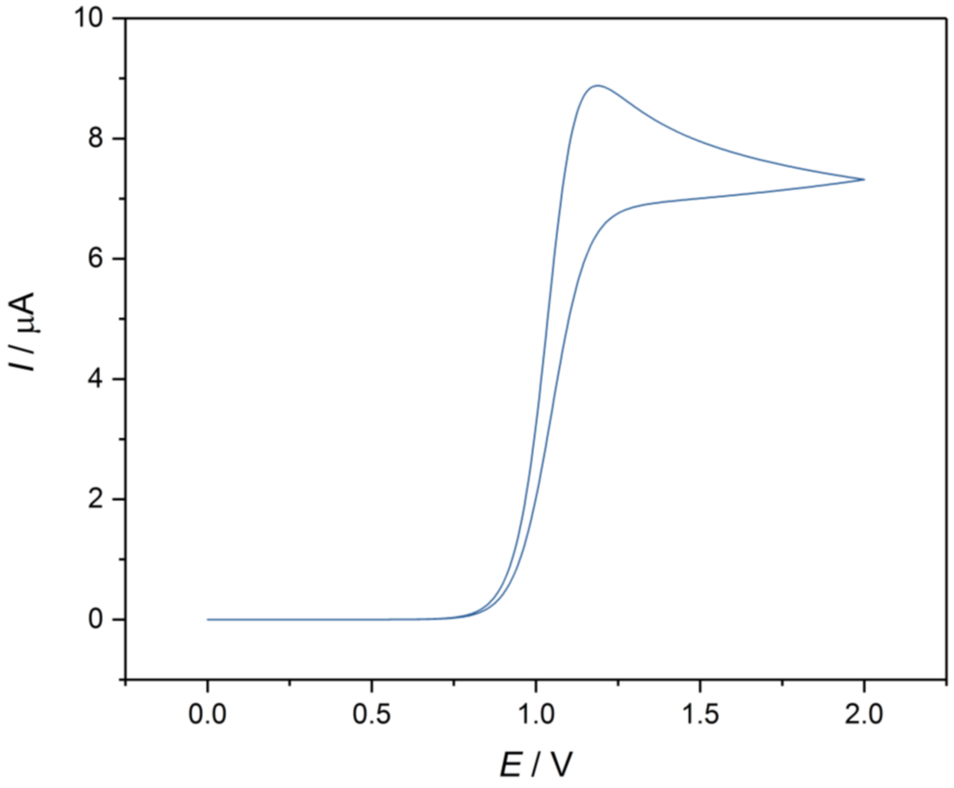

Figure S8. Simulated voltammetric response for the oxidation of bromide to bromine.

S9: Simulation of Double Potential Step Chronoamperometry

The silver nanoparticle oxidation at the carbon fibre electrode in the bromide solution is simulated for the double-potential chronoamperometry experiment. The carbon fibre attached at the substrate is approximated as a hemicylinder model and thus only the concentration variation in one dimension, the direction along the hemicylinder radius r, needs to be taken into consideration. In the double-potential chronoamperometry experiment, Br^- is oxidized to Br_2 at the first potential step E_1 and Br_2 is reduced to Br^- at the second potential step E_2. We assume the surface concentrations of the Br_2/Br^- redox couple are determined by the Nernst equation and are estimated from the experiment current. At both electrode potentials, the silver nanoparticle can be oxidized at the electrode surface. AgNPs in the solution can be also oxidized by Br_2 produced via the redox reaction during the first potential step.

$$\mathrm{Br^-} - \mathrm{e^-} \rightarrow \frac{1}{2}\mathrm{Br_2} \qquad \text{(S1)}$$

$$\mathrm{AgNP} - n_{\mathrm{Ag}}e^- \rightarrow n_{\mathrm{Ag}}\mathrm{Ag^+} \qquad \text{(S2)}$$

$$\mathrm{AgNP} + \frac{n_{\mathrm{Ag}}}{2}\mathrm{Br_2} \rightarrow n_{\mathrm{Ag}}\mathrm{AgBr} \qquad \text{(S3)}$$

$$c_{\mathrm{Br_2}}(r = r_{\mathrm{el}}) = const \times c^*_{\mathrm{Br^-}}, c_{\mathrm{Br^-}}(r = r_{\mathrm{el}}) = (1 - 2 \times const)c^*_{\mathrm{Br^-}},\ at\ E_1 \qquad \text{(S4)}$$

$$c_{\mathrm{Br_2}}(r = r_{\mathrm{el}}) = 0, c_{\mathrm{Br^-}}(r = r_{\mathrm{el}}) = c^*_{\mathrm{Br^-}}, at\ E_2 \qquad \text{(S5)}$$

$$c_{\mathrm{AgNP}}(r = r_{\mathrm{el}}) = 0 \qquad \text{(S6)}$$

where $c^*_{\mathrm{Br^-}}$ is the bulk concentration ($r \rightarrow \infty$) of Br^- and $c(r=r_{\mathrm{el}})$ refers to the surface concentration at the hemicylinder electrode with the radius of r_{el}. Before the redox reaction, there is only Br^- in solution and the surface concentration of Br^- equals $c^*_{\mathrm{Br^-}}$. After applying a potential, the surface concentration varies as a function of potential. At the first potential step E_1 in the double-potential chronoamperometry, a small portion of Br^- is oxidized to be Br_2. In equation (7), *const* = 0.005 is a fitting parameter, which is not calculated from any assumption of the electron transfer kinetics but directly determined by fitting to the current of the first potential step in Figure 4d. At the second potential E_2, Br_2 is fully reduced to Br^- at the electrode and the surface concentration of Br_2 is therefore zero. At both electrode potentials, the AgNPs can be oxidized at the electrode surface.

The reaction is simplified as an irreversible second-order homogeneous chemical reaction with a rate constant k_{sol} ($\mathrm{mM^{-1}\ s^{-1}}$). The mass transport of AgNP, Br^- and Br_2 is described by the diffusion equation, Fick's second law, combined with the chemical reaction between AgNP and Br_2.

$$\frac{\partial c_{\mathrm{Br_2}}}{\partial t} = D_{\mathrm{Br_2}} \nabla^2 c_{\mathrm{Br_2}} - \frac{n_{\mathrm{Ag}}}{2} k_{\mathrm{sol}} c_{\mathrm{Br_2}} c_{\mathrm{AgNP}} \quad \text{(S7)}$$

$$\frac{\partial c_{\mathrm{Br^-}}}{\partial t} = D_{\mathrm{Br^-}} \nabla^2 c_{\mathrm{Br^-}} \quad \text{(S8)}$$

$$\frac{\partial c_{\mathrm{AgNP}}}{\partial t} = D_{\mathrm{AgNP}} \nabla^2 c_{\mathrm{AgNP}} - k_{\mathrm{sol}} c_{\mathrm{Br_2}} c_{\mathrm{AgNP}} \quad \text{(S9)}$$

Defining the parameter t as the reaction time, $c*$ as the concentration of AgNPs in bulk solution and D as the diffusion coefficient, the parameters used in the simulation are identical with the experimental conditions, as follows: c^*_{AgNP}= 1.2x10^{-9} mM, $c^*_{Br^-}$= 20 mM, D_{AgNP} = 1.0x10^{-11} m^2 s^{-1}, $D_{\mathrm{Br-}}$ = D_{Br2} = 1.0 x10^{-9} m^2 s^{-1}, n_{Ag} is the average amount of silver atoms in one nanoparticle and equal to 2.2*10^6. $n_{\mathrm{Ag}}/2$ is the amount of Br_2 molecules needed to oxidize one silver nanoparticle. The boundary conditions and equations used are given as follows:

$t = 0, r \qquad c_{\mathrm{AgNP}} = c^*_{\mathrm{AgNP}},\ c_{\mathrm{Br^-}} = c^*_{\mathrm{Br^-}},\ c_{\mathrm{Br_2}} = 0.$

$r = r_{el}, t \qquad c_{\mathrm{AgNP}} = 0,\ c_{\mathrm{Br^-}} = 0.99 c^*_{\mathrm{Br^-}},\ c_{\mathrm{Br_2}} = 0.005 c^*_{\mathrm{Br^-}}$ at E_1.

$c_{\mathrm{AgNP}} = 0,\ c_{\mathrm{Br^-}} = c^*_{\mathrm{Br^-}},\ c_{\mathrm{Br_2}} = 0$ at E_2.

$r > r_{el}, t$

$$\frac{\partial c_{\mathrm{AgNP}}}{\partial t} = D_{\mathrm{AgNP}} \left(\frac{\partial^2 c}{\partial r^2} + \frac{1}{r}\frac{\partial c}{\partial r} \right) - k_{\mathrm{sol}} c_{\mathrm{AgNP}} c_{\mathrm{Br_2}}$$

$$\frac{\partial c_{\mathrm{Br^-}}}{\partial t} = D_{\mathrm{Br^-}} \left(\frac{\partial^2 c}{\partial r^2} + \frac{1}{r}\frac{\partial c}{\partial r} \right)$$

$$\frac{\partial c_{\mathrm{Br_2}}}{\partial t} = D_{\mathrm{Br_2}} \left(\frac{\partial^2 c}{\partial r^2} + \frac{1}{r}\frac{\partial c}{\partial r} \right) - \left(\frac{n_{\mathrm{Ag}}}{2} \right) k_{\mathrm{sol}} c_{\mathrm{AgNP}} c_{\mathrm{Br_2}}$$

$r = r_{\max}, t \qquad c_{\mathrm{AgNP}} = c^*_{\mathrm{AgNP}},\ c_{\mathrm{Br^-}} = c^*_{\mathrm{Br^-}},\ c_{\mathrm{Br_2}} = 0.$

The mass transport equation is numerically solved by the finite difference method. The simulation program is written in Matlab R2017a and run on an Intel(R) Xeon(R) 3.60G CPU.

S10: Concentration Profiles from Simulated Data

Figure S10a presents the experimentally determined concentration profile against three simulated profiles fitted with varying rate constants. The profile most representative of the experimental results (c), 30 mM^{-1} s^{-1}, is presented in the main text. This is derived from considering the half-wave (distance at C =0.5) profiles, presented in Figure S10b. The half-wave distances are initially comparable to the simulated results with a rate constant of 30 mM^{-1} s^{-1} but drop off much faster, tending to a constant value whilst the simulation predicts that the half-wave distance continues to increase.

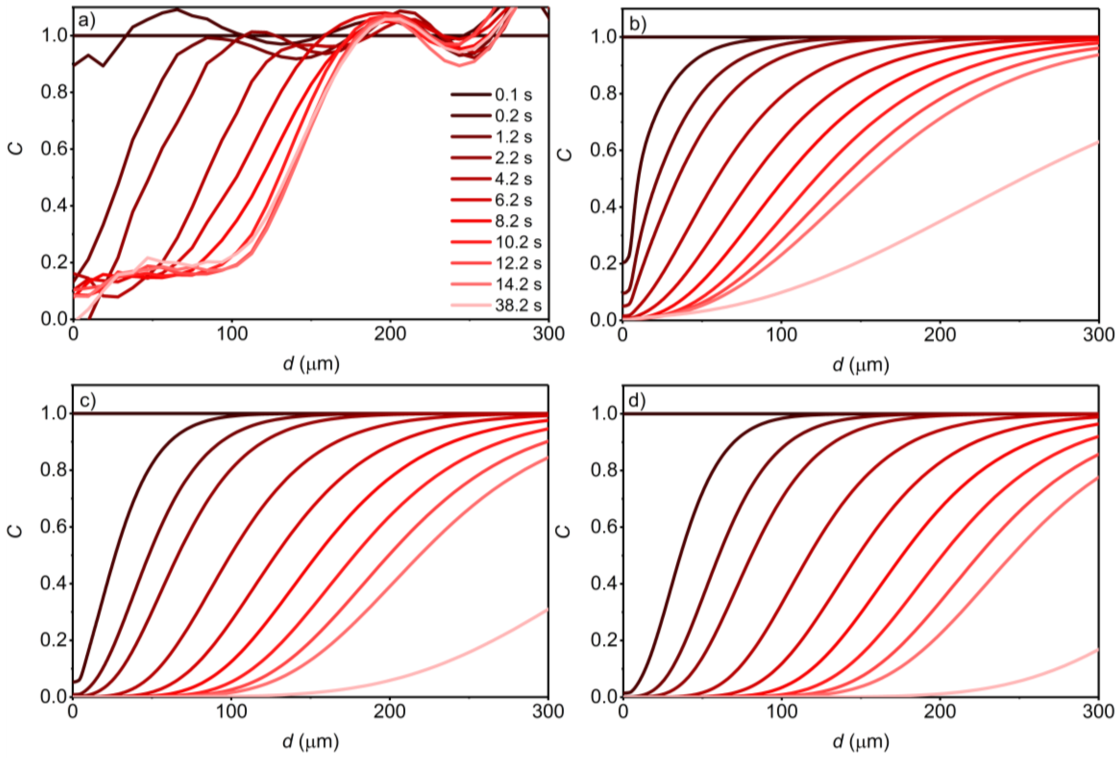

Figure S10a. (a) Experimentally calculated concentration profile for 1.2 pM AgNPs in 20 mM KBr as a function of time. (b), (c), and (d) show equivalent simulated profiles assuming a rate constant of k_{sol} = 10 mM^{-1} s^{-1}, 30 mM^{-1} s^{-1}, and 50 mM^{-1} s^{-1} respectively.

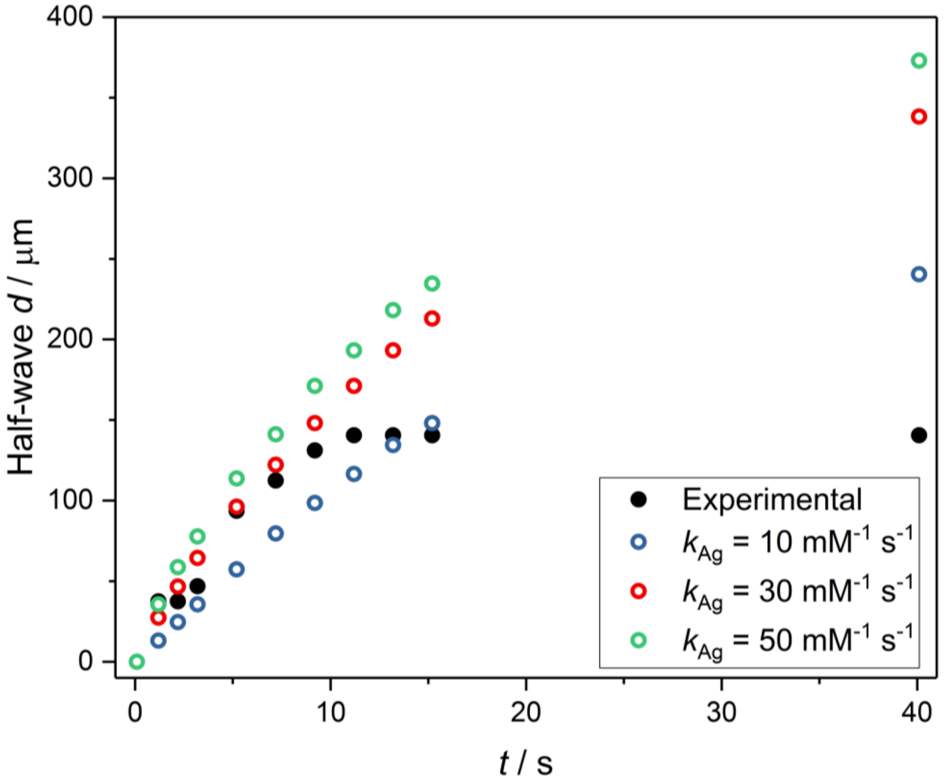

Figure S10b. Half-wave distances for the experimentally determined and simulated concentration profiles (black solid circles and open circles respectively).

References

1. Esteban, R.; Taylor, R. W.; Baumberg, J. J.; Aizpurua, J., How Chain Plasmons Govern the Optical Response in Strongly Interacting Self-Assembled Metallic Clusters of Nanoparticles. *Langmuir* **2012**, *28*, 8881-8890.
2. Weast, R. C., *Crc Handbook of Chemistry and Physics: 1974-1975 (55th Ed.)*; CRC Press, 1974.
3. Xu, J.; Georgescu, N. S.; Scherson, D. A., The Oxidation of Bromide on Platinum Electrodes in Aqueous Acidic Solutions: Electrochemical and in Situ Spectroscopic Studies. *J. Electrochem. Soc.* **2014**, *161*, H392-H398.
